# *Mycoplasma bovis* MBOV_RS02825 Encodes a Secretory Nuclease Associated with Cytotoxicity

**DOI:** 10.3390/ijms17050628

**Published:** 2016-04-29

**Authors:** Hui Zhang, Gang Zhao, Yusi Guo, Harish Menghwar, Yingyu Chen, Huanchun Chen, Aizhen Guo

**Affiliations:** 1 The State Key Laboratory of Agricultural Microbiology, Huazhong Agricultural University, Wuhan 430070, China; dkyzhanghui@163.com (H.Z.); zhaog0524@163.com (G.Z.); guoyusi0626@163.com (Y.G.); drharishsanja@hotmail.com (H.M.); chenyingyu@mail.hzau.edu.cn (Y.C.); chenhch@mail.hzau.edu.cn (H.C.); 2 College of Veterinary Medicine, Huazhong Agricultural University, Wuhan 430070, China; 3 Key Laboratory of Development of Veterinary Diagnostic Products, Ministry of Agriculture, Wuhan 430070, China; 4 Hubei International Scientific and Technological Cooperation Base of Veterinary Epidemiology, Huazhong Agricultural University, Wuhan 430070, China

**Keywords:** MBOV_RS02825, *Mycoplasma bovis*, nuclease, secretory protein, TNASE_3 domain, neutrophil extracellular traps (NETs), apoptosis, binding and internalization

## Abstract

This study aimed to determine the activity of one *Mycoplasma bovis* nuclease encoded by MBOV_RS02825 and its association with cytotoxicity. The bioinformatics analysis predicted that it encodes a Ca^2+^-dependent nuclease based on existence of enzymatic sites in a TNASE_3 domain derived from a *Staphylococcus aureus* thermonuclease (SNc). We cloned and purified the recombinant MbovNase (rMbovNase), and demonstrated its nuclease activity by digesting bovine macrophage linear DNA and RNA, and closed circular plasmid DNA in the presence of 10 mM Ca^2+^ at 22–65 °C. In addition, this MbovNase was localized in membrane and rMbovNase able to degrade DNA matrix of neutrophil extracellular traps (NETs). When incubated with macrophages, rMbovNase bound to and invaded the cells localizing to both the cytoplasm and nuclei. These cells experienced apoptosis and the viability was significantly reduced. The apoptosis was confirmed by activated expression of phosphorylated NF-κB p65 and Bax, and inhibition of Iκβα and Bcl-2. In contrast, rMbovNase^Δ181–342^ without TNASE_3 domain exhibited deficiency in all the biological functions. Furthermore, rMbovNase was also demonstrated to be secreted. In conclusion, it is a first report that MbovNase is an active nuclease, both secretory and membrane protein with ability to degrade NETs and induce apoptosis.

## 1. Introduction

Since its discovery in 1961 in the USA, *Mycoplasma bovis* has become recognized as a major pathogen in cattle [1], causing respiratory disease, mastitis, arthritis, and a variety of other diseases in both beef and dairy cattle worldwide [2]. *M. bovis* was first reported in China in 2008 as a cause of pneumonia and arthritis associated with over 80% morbidity and 10% mortality on average in feedlot store cattle [3]. Measures to prevent and treat *M. bovis* infection are limited by lack of knowledge of its pathogenesis.

Nucleases are important constituents of mycoplasmal membranes involved in the acquisition of host nucleic acids required for growth [4]. Homologs of thermostable bacterial nucleases had been identified with several mycoplasmas species [5,6,7]. Moreover, several nucleases have been implicated in mycoplasma-mediated host pathogenicity and cytotoxicity [7,8,9]. Based on the sequence alignment, there is a highly conserved region named TNASE_3 belonging to micrococcal nuclease (thermonuclease) COG1525 [10]. The conserved region has several catalytic sites involved in the nuclease activity and binding of calcium ions [11]. This region of *Mycoplasma gallisepticum* MGA_0676 was associated with nuclear translocation and apoptosis of chicken cells [7]. In addition, bacterial nucleases also contribute to breaking down the DNA backbone of neutrophil extracellular traps (NETs) which are produced by activated neutrophils at sites of infection. NETs contain nuclear or mitochondrial DNA with embedded antimicrobial peptides, histones, and cell specific proteases, and thereby provide an extracellular matrix to entrap and kill various microbes [12,13]. However, the biological functions of *M. bovis* nucleases are not yet clear. The possible pathogenic effect on the interaction between *M. bovis* nucleases and host cells is worth investigating.

In our previous study (data not published), we identified an immunogenic membrane protein encoded by MBOV_RS02825, which is a nuclease homologue by two-dimensional (2-D) electrophoresis and its MALDI-TOF MS spectrum. However, its possible nuclease activity and pathogenic effect has not yet been examined. Therefore, this study aimed to determine the enzymatic activity and possible association with pathogenic effect. The evidences demonstrated that this protein (MbovNase) is an active nuclease, and the first secretory protein ever identified for *M. bovis*. Besides, it can bind to and enter bovine macrophages to induce cytotoxic effects and apoptosis.

## 2. Results

### 2.1. MBOV_RS02825 Sequence Has a Typical Nuclease Structure

**Figure 1:**
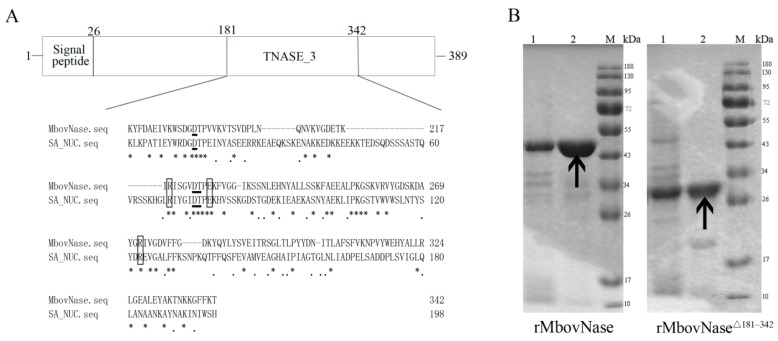
MbovNase organization, expression, and purification. (**A**) Schematic of MbovNase. The protein contains 389 amino acids with N terminal type 1 signal sequence (aa 1–26) inserting into the membrane. Alignment of amino acid sequences in *Staphylococcus aureus* thermonuclease enzymatic domain (SNc) shows that amino acids essential to nuclease activity arginine (R) residues at 219 and 272 and glutamic acid (E) at 227 (shown by the open boxes), and the aspartic acid (D) at aa 194, 224 and threonine (T) at aa 225 for the calcium binding motif (shown with the underline mark) are conserved sites [11]; Asterisks indicate positions conserved and dots represent positions similar; (**B**) Detection of expression of rMbovNase and its variant by 12% SDS-PAGE. The proteins were stained with coomassie brilliant blue R250 after SDS-PAGE. **Left**: His-tagged rMbovNase; **Right**: His-tagged rM.bovNase^Δ181–342^. Lane 1: Total-cell lysate of *E. coli* pET30a-MBOV_RS02825 (**left**) or *E. coli* pET30a-MBOV_RS02825^Δ181–342^ (**right**) induced by IPTG; Lane 2: Purified His-tagged rMbovNase (**left**) and rMbovNase^Δ181–342^ (**right**). The arrows indicate rMbovNase and rMbovNase^Δ181–342^. *M*w (kDa) represents molecular weight of reference proteins.

Based on its genome sequence, it was predicted that MBOV_RS02825 of *M. bovis* HB0801 encodes MbovNase, a 44.2 kDa protein with 389 amino acids (aa) and an isoelectric point (pI) of 8.16. Using a hidden Markov model (TMHMM)2.0 [14] and LipoP 1.0 [15] to predict the transmembrane and signal peptidase type indicated this is a peripheral membrane protein with an N terminal (aa 1–26) type I signal sequence inserting into the membrane, with C terminal stretching out, and a typical cysteine cleavage site at 26th residue by SignalP 4.0 [16]. The fragment spanning aa 181–342 is homologous to the SNc region of *S. aureus* thermonuclease (SA_NUC). Although the aa similarity between the SA_NUC and TNASE_3 regions of MbovNase is only 24.75%, it was predicted by prosite analysis that MbovNase contains the amino acids necessary for nuclease activity including arginine (R) at residues 219 and 272, glutamic acid (E) at residue 227, a conserved Ca^2+^ activated motif characterized by two aspartates (D) at positions 194 and 224, and one threonine (T) at position 225 (Figure 1A). These residues are speculated to be important based on the crystal structure of *S. aureus* thermonuclease (PDB ID: 1SNQ) (Figure 1A) [11].

### 2.2. Expression of rMbovNase in Escherichia coli

To determine whether MBOV_RS02825 encodes a functional nuclease, the TGA codon was changed into TGG by site-directed PCR mutagenesis at six nt positions (334, 448, 493, 874, 967, 1009) in *MBOV_RS02825* to ensure that tryptophan was encoded in *E. coli*. The whole gene with these six site mutations and without the signal sequence was PCR-amplified and cloned into pET30a. The inserted fragment was confirmed by DNA sequencing. The recombinant His-tagged N-terminal rMbovNase protein was expressed by *E. coli*. The molecular mass was estimated by SDS-PAGE to be about 48 kDa resulting from 44 kDa protein plus a 4 kDa tag of 6 histidines, as expected (Figure 1B). The purity of rMbovNase is about 95%, while that of rMbovNase^Δ181–342^ is about 80%. This purified rMbovNase and rMbovNase^Δ181–342^ in PBS (pH 7.4) was stable at −80 °C for at least three months shown by less than 5% reduction in concentrations (rMbovNase from 2597.239 to 2505.020 μg·mL^−1^, while rMbovNase^Δ181–342^ from 1345.252 to 1323.454 μg·mL^−1^).

Mice were immunized with purified rMbovNase and its antiserum with an ELISA titer of 2 × 10^5^ was produced 2 weeks after the third exposure. To evaluate the biological effects of the rMbovNase TNASE_3 region, we successfully constructed a mutated gene with a deletion of this functional domain. As predicted, the rMbovNase^Δ181–342^ variant was shown by SDS-PAGE to be about 30 kDa including protein size of about 26 kDa plus a 4 kDa tag of 6 histidines (Figure 1B).

### 2.3. Determinants of rMbovNase Nuclease Activity

**Figure 2:**
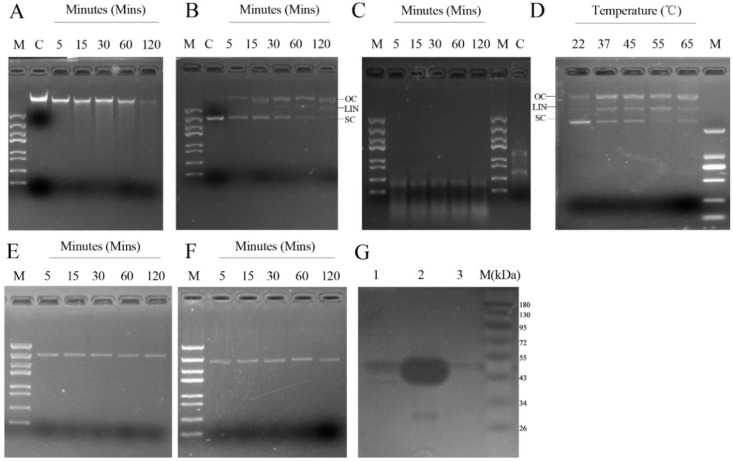
Assay of rMbovNase nuclease activity. **A**–**C**: Different substrates, 1 µg BoMac cellular DNA (**A**); plasmid DNA (**B**); BoMac cellular RNA (**C**), incubated with 2.5 µg rMbovNase plus 10 mM CaCl_2_ in 100 mM Tris-HCl buffer (pH 8.5). Samples were collected at 5, 15, 30, 60, and 120 min and reactions were stopped by adding 10 mM EDTA and resolved on 1% agarose gels. Untreated DNA and RNA were negative controls (Lane C). Open-circle (OC), linear (LIN), and supercoiled (SC) forms of plasmid DNA are indicated; (**D**) rMbovNase had nuclease activity with plasmid DNA at temperatures from 22 to 65 °C. E: Nuclease activity of rMbovNase^Δ181–342^. 2.5 µg rMbovNase^Δ181–342^ (**E**) was incubated with plasmid DNA (1 μg) in the presence of 10 mM CaCl_2_ for 5, 15, 30, 60, or 120 min; PBS (**F**) was used as a negative control; (**G**) Zymogram analysis of nuclease activity using 12% SDS-PAGE gels containing 160 μg·mL^−1^ of herring sperm DNA in renaturation buffer. Lane 1, Total *M. bovis* cell lysate protein; Lane 2, Purified rMbovNase; Lane 3, Purified rMbovNase^Δ181–342^.

Since MBOV_RS02825 has a conserved Ca^2+^-activated motif (Figure 1A), the Ca^2+^ requirement for rMbovNase nuclease activity was tested. In the presence of 10 mM Ca^2+^, rMbovNase had nuclease activity, shown by digesting DNA from bovine macrophage (BoMac) cells (Figure 2A), a circular plasmid DNA (pET30a) (Figure 2B) and BoMac cellular RNA (Figure 2C) in a time-dependent manner. Compared to DNA, rMbovNase degraded RNA more efficiently, such as finishing digestion to the similar concentrations of substrates in a shorter time (5 min). Its thermostablility, conferred by the themonuclease motif, was confirmed by the finding that rMbovNase could cut supercoiled plasmid DNA into three bands at a wide range of temperatures from 22 to 65 °C. The activity increased with temperature (Figure 2D). To determine the role of the TNASE_3 region in rMbovNase nuclease activity, we tested the ability of rMbovNase^Δ181–342^ variant to digest plasmid DNA. Unlike rMbovNase (Figure 2B), the variant (Figure 2E) could not degrade plasmid DNA, similar to PBS (Figure 2F), which was a negative control. In the zymogram analysis, mycoplasma whole-cell lysate produced a clear band of approximately 44 kDa for nature form of MbovNase (Figure 2G, Lane 1) and rMbovNase produced a very intense band about 48 kDa (Figure 2G, Lane 2) due to the degradation of herring sperm DNA present in the SDS-PAGE gel. No rMbovNase^Δ181–342^ band about 30 kDa was observed in the gels (Figure 2G, Lane 3). These results indicate that both mycoplasma MbovNase and rMbovNase were very active, and that the nuclease activity was determined by the TNASE_3 region.

The effect of metal ions on rMbovNase nuclease activity was tested using plasmid DNA as the substrate. CaCl_2_ enhanced rMbovNase activity, with an optimum concentration of 10 mM based on the degradation effect on both open circle and supercoiled fraction of DNA (Figure S1A). The addition of other metal ions including MgCl_2_, MnCl_2_, ZnCl_2_, KCl, and NaCl did not stimulate rMbovNase to the extent observed for CaCl_2_ (Figure S1B–F).

### 2.4. rMbovNase Breakdown of NETs

**Figure 3:**
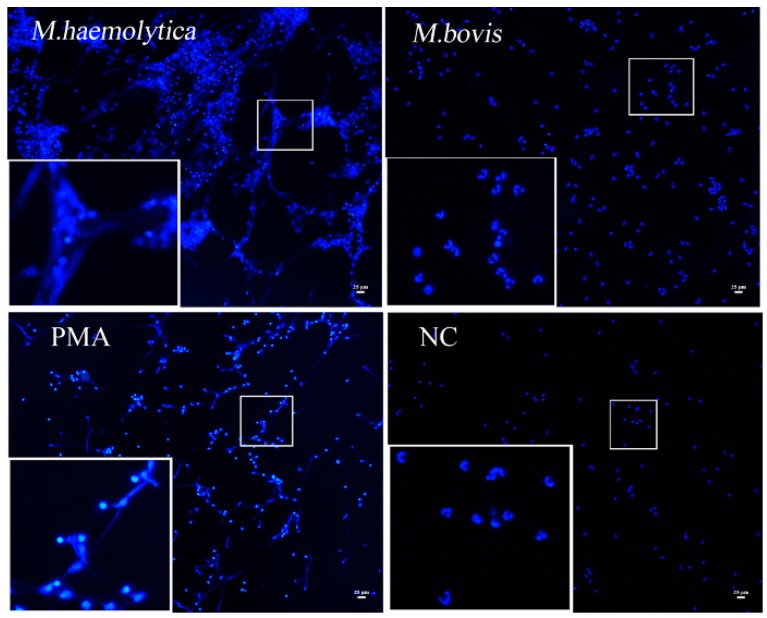
Neutrophil extracellular traps (NETs) formation induced by different stimulators. NETs were induced by *Mannheimia haemolytica* (*M. haemolytica*) (MOI = 100) and 200 nM phorbol 12-myristate 13-acetate (PMA), but not by *M. bovis* HB0801 strain (MOI = 1000). PBS treatment was taken as the negative control. Extra- and intracellular DNA was stained blue by DAPI (magnifications: ×100; the zoomed images in the lower left boxes are ×3 magnifications of the images in middle small boxes).

To directly observe whether *M. bovis* infection was able to induce NETs, the bovine neutrophils were stimulated by 200 nM PMA or *Mannheimia haemolytica* (*M. haemolytica*) at a multiplicity of infection (MOI) of 100, or *M. bovis* strain HB0801 at different MOI (10, 100, 1000) for different times (1, 2, 3, and 4 h) and observed by a fluorescence microscopy. Unlike *M. haemolytica* and PMA stimulation, *M. bovis* strain HB0801 itself under all conditions could not generate NETs even at a MOI as high as 1:1000 (Figure 3).

**Figure 4:**
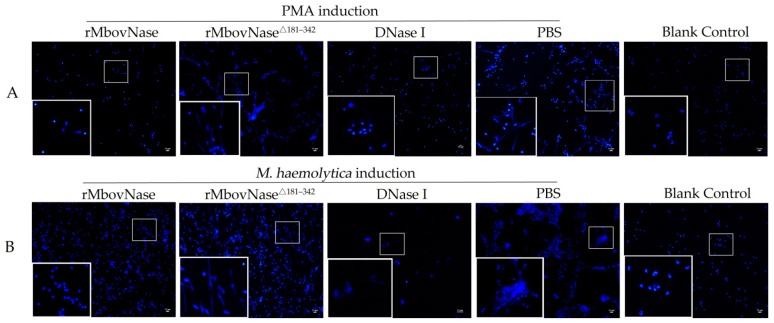
NETs degradation by rMbovNase and its variant rMbovNase^Δ181–342^. (**A**) NETs were generated by PMA induction; (**B**) NETs were induced by *M. haemolytica*. The rMbovNase and the variant, together with DNase I (positive control) and PBS (negative control) were added into activated neutrophils in both A and B, and only rMbovNase and DNase I apparently degraded NETs, while the variant and PBS did not. No treatment was performed in blank control. The fields in the small squares are zoomed in the large squares (magnifications: ×100; the zoomed images in the lower left boxes are ×3 magnifications of the images in middle small boxes).

The DNA matrix, which was the backbone of NETs structures, was visualized by DAPI staining. To investigate the differential effect of rMbovNase and its variant rMbovNase^Δ181–342^ on NETs, the neutrophils activated by PMA and *M. haemolytica* were exposed to rMbovNase or the variant for an additional 90 min (Figure 4). The nuclei and DNA fibers released from NETs could be observed clearly after DAPI staining. However, after addition of rMbovNase, the amount of observable DNA fibers in NETs induced by both PMA and *M. haemolytica* was reduced, like the commercial DNase (40 U per well) positive control. In contrast, addition of neither rMbovNase^Δ181–342^ nor PBS did not cause apparent reduction on the DNA fibers in NETs. Meanwhile, the neutrophils in blank control did not develop NETs (Figure 4).

### 2.5. Localization of MbovNase in M. bovis Cells

**Figure 5:**
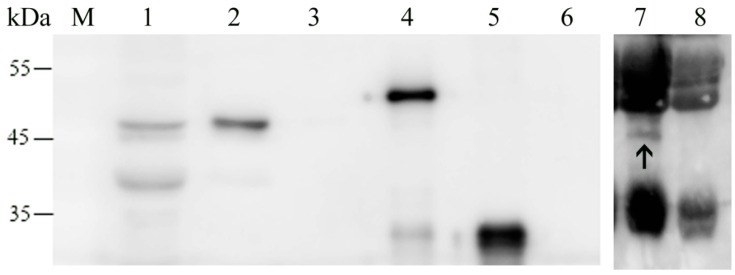
Localization of MbovNase in *M. bovis* cells detected with western blot analysis. The different fractions of *M. bovis* cells, culture supernatant and the purified rMbovNase or rMbovNase^Δ181–342^ were subjected to SDS-PAGE and western blot analysis with mouse antiserum to rMbovNase. MbovNase is not only present in total *M. bovis* cell protein (Lane 1) and membrane fraction (Lane 2), but also in culture supernatant as a secretory protein (shown by the arrow in lane 7). However, it was not in the cytoplasmic fraction (Lane 3). The rMbovNase (Lane 4) is 4 kDa larger than the nature MbovNase due to 6 ×His tag, while rMbovNase^Δ181–342^ (Lane 5) is about 30 kDa, 18 kDa smaller due to the deletion of TNASE_3 domain. The pure bovine serum albumin (Lane 6) does not produce any band, while the media with many nonspecific proteins from horse serum and yeast extract display nonspecific bands in lane 7 and 8, however there is no target band in lane 8 as shown by the arrow in lane 7. Molecular weight of the reference proteins (kDa) is indicated on the left.

To detect the location of rMbovNase in *M. bovis*, *M. bovis* whole proteins, cytoplasmic fractions, and membrane fractions extracts, the concentrated culture supernatants were prepared, meanwhile the purified rMbovNase and its variant were used as positive controls, with bovine serum albumin and growth media of *M. bovis* as negative controls. Each kind of samples was loaded 50 ng in 10 μL and subjected to SDS-PAGE and western blot assay. We found that rMbovNase was localized in the cell membrane and culture supernatant rather than cytoplasmic fraction, confirming that it is an amphoteric protein with characteristics of both membrane-location (Figure 5, Lane 2) and secretion (Figure 5, Lane 7). In addition, antiserum to rMbovNase recognized both rMbovNase (Figure 5, Lane 4) and its variant (Figure 5, Lane 5) suggesting that although deletion of SNc region removed enzyme activity, it did not eliminate rMbovNase immunogenicity. The size of immature nature protein in the membrane is about 44 kDa, and that of mature protein in supernatant fractions a little smaller due to the cleaved signal peptide which size is 2.7 kDa, while that of *rMbovNase* and its variant was 48 kDa and 30 kDa respectively (Figure 5).

### 2.6. TNASE_3 Is Essential for rMbovNase Binding and Internalization in BoMac Cells

**Figure 6:**
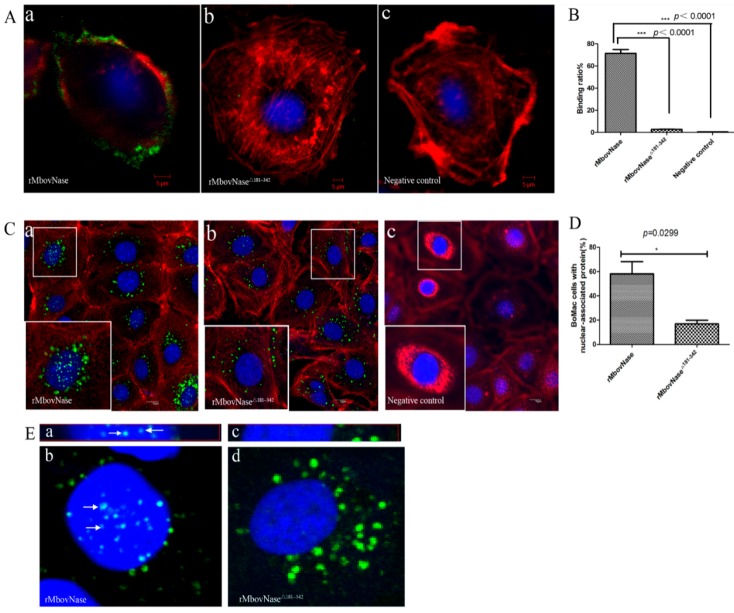
Assay of rMbovNase adhesion and invasion to BoMac cells. (**A**) Binding of rMbovNase and its variant to BoMac cells observed by confocal laser microscopy. BoMac cells were incubated with 10 μg·mL^−1^ rMbovNase (**a**) or rMbovNase^Δ181–342^ (**b**) for 30 min at 4 °C. The attached protein was probed with mouse antiserum against rMbovNase (1:500) and then with goat anti-mouse IgG conjugated with FITC (green) Cellular actin filaments and nuclei were stained with rhodamine phalloidin (red) and DAPI (blue) respectively; magnifications: ×1000; (**B**) Binding assayed by flow cytometry. Standard deviations from the individual measurements are indicated as bars. *** *p* < 0.01; (**C**) Effect of TNASE_3 domain on invasion of rMbovNase (**a**) and rMbovNase^Δ181–342^ (**b**). The method was the same as in the binding assay but incubated for 24 h at 37 °C. Laser confocal microscopy shows the deletion of TNASE_3 region in rMbovNase impaired its nuclear translocation but did not effect its internalization to cytoplasm. PBS treatment was negative control (**c**); magnifications: ×400; the zoomed images in the lower left boxes are ×3 magnifications of the images in middle small boxes; (**D**) Percentage of BoMac cells with nuclear-associated rMbovNase and rMbovNase^Δ181–342^ immunofluorescence 24 h post-infection. Values are the means of 10 random microscopic fields and are representative of two independent experiments. Results were expressed as mean percentages ± SEM and were analyzed with the unpaired *t*-test. The difference between rMbovNase and its variant was statistically significant (*p = 0.029); (**E**) Localization of rMbovNase (**a**,**b**) in nuclei of BoMac cells shown by *Z*-series scanning. White arrows indicate the localization of rMbovNase within nuclear regions based on serially produced sections, while rMbovNase^Δ181–342^ (**c**,**d**) only appeared in cytoplasm; magnifications: ×1000.

To evaluate involvement of TNASE_3 region in rMbovNase biological function, we detected the binding and internalization of rMbovNase and its variant with deletion of TNASE_3 region to BoMac cells using the indirect immunofluorescence technique. As shown in Figure 6A, rMbovNase protein (green) strongly bound to BoMac cells at 4 °C for 30 min, surrounding the cell along the cell membrane; only a small portion of the protein was internalized as shown by merged yellow and orange (Figure 6A(a)). However, a little binding (green) was seen with either rMbovNase^Δ181–342^ (Figure 6A(b)) or PBS (Figure 6A(c)) treatment. Quantitative assay using flow cytometry found that the binding ratio of rMbovNase-treated was 71.3% ± 3.5%, and a significantly smaller ratio of 2.77% ± 0.22% in rMbovNase^Δ181–342^ treated cells and background level of 0.51% ± 0.07% in PBS control (Figure 6B). The difference among binding ratios was very significant (*p* < 0.001). These findings clearly indicated that TNASE_3 region is associated with efficient binding to BoMac cells.

As shown in Figure 6C, rMbovNase (Figure 6C(a)) was internalized and distributed among the cytoplasm, perinuclear region, and within the nuclei of the cells after co-incubation of protein and cells for 24 h at 37 °C. In contrast, the rMbovNase^Δ181–342^ was mainly observed in the cytoplasmic and infrequently in BoMac nuclei (Figure 6C(b)). No green signal was seen in PBS treated cells (Figure 6C(c)). The frequency of nuclear localization of rMbovNase and rMbovNase^Δ181–342^ was determined with ImagePro Plus 6.0 software [17] by measuring positive cells with protein nuclear distribution in 10 random fields from each group. Then the ratio (%) of positive cells to the total number of cells in the same fields was calculated, which was 58% for wild type protein and 16% for the variant, respectively, and the difference between them was significant (*p* < 0.05) (Figure 6D). We further chose one of cross and vertical section images, respectively, from the apical, central, and basal planes of nuclei of BoMac cells to further demonstrate the internalization of rMbovNase (Figure 6E). The images clearly showed obvious co-localization of rMbovNase (Figure 6E(a,b)), rather than rMbovNase^Δ181–342^ (Figure 6E(c,d)) within nuclei.

### 2.7. rMbovNase-Induced Cytotoxicity and Apoptosis of BoMac Cells

**Figure 7:**
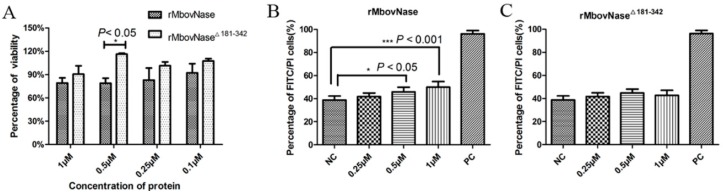
Cytotoxic effect of rMbovNase on BoMac cells. (**A**) BoMac cells treated with rMbovNase or rMbovNase^Δ181–342^ proteins for 24 h at 37 °C, and cell viability was determined by MTT assay. Flow cytometry assay on BoMac cells treated with rMbovNase (**B**) and rMbovNase^Δ181–342^ (**C**) protein for 24 h and stained with Annexin V and propidium iodide (PI). PBS treatment was taken as a negative control (NC); apoptosis inducer from the commercial kit was a positive control (PC). * and *** represent *p* < 0.05 and 0.001 respectively.

The differential cytotoxic effects of rMbovNase and rMbovNase^Δ181–342^ on BoMac cells were investigated by the methylthiazol tetrazolium (MTT) assay. As shown in Figure 7A, compared with rMbovNase^Δ181–342^, rMbovNase caused an approximately 10%–20% reduction in the viability of BoMac cells. The difference was most significant (*p* < 0.05) at a concentration of 0.5 μM.

Apoptosis of BoMac cells induced by both proteins was assayed by Annexin V/PI staining and flow cytometry detection. The rMbovNase induced apoptosis in a concentration-dependent manner. The difference in percentages of apoptotic cells in rMbovNase-treated cells and negative control was significant at the *p* < 0.05 level at 0.5 µM and *p* < 0.001 at 1 µM rMbovNase (Figure 7B). However rMbovNase^Δ181–342^ did not induce a significantly high percentage of apoptotic BoMac cells at any of the tested concentrations compared to the negative control (Figure 7C).

### 2.8. Expression of Some Signal Molecules Associated with Apoptosis

**Figure 8:**
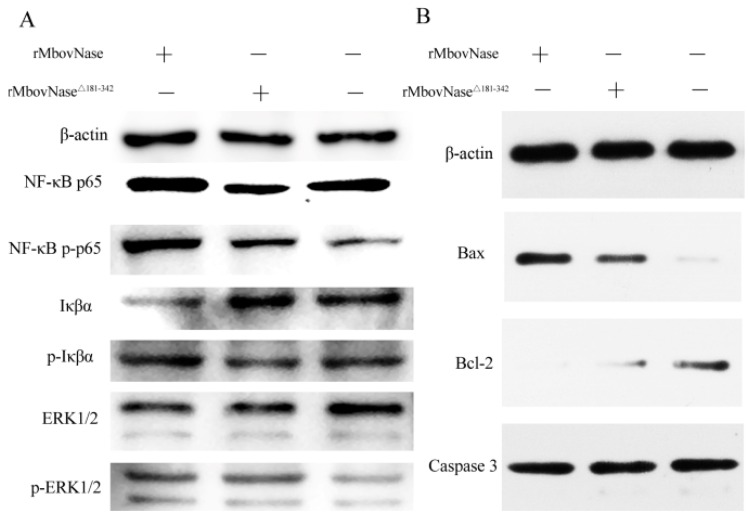
Effects of rMbovNase and rMbovNase^Δ181–342^ on expression of NF-κB p65, MAPK ERK1/2, Bax, Bcl-2 and Caspase 3 in BoMac cells. (**A**) The expression of signal transduction molecules associated with NF-κB signal transduction pathway; (**B**) The expression of apoptosis-related proteins. Western blot analysis of total protein extracts was performed for each group after 1 μM rMbovNase or rMbovNase^Δ181–342^ treatment at 37 °C for 24 h. Commercial antibodies specific to the listed proteins were used, while β-actin was taken as the internal reference.

To further elucidate the mechanisms of apoptosis signal transduction pathways, the expression of phosphorylated NF-κB p65, Iκβα, and ERK 1/2 signal transduction molecules and the apoptosis-related proteins Bax, Bcl-2, and Caspase 3 in BoMac cells was analyzed with western blot. The results showed that rMbovNase enhanced expression of phosphorylated NF-κB p65, pro-apoptotic Bax protein, while it inhibited expression of Iκβα, anti-apoptotic Bcl-2 protein compared with rMbovNase^Δ181–342^, but not ERK 1/2 and its phosphorylated form. Neither rMbovNase nor its variant significantly changed Caspase 3 expression (Figure 8A,B).

## 3. Discussion

In the present study, we firstly found an *M. bovis* nuclease rMbovNase, a both membrane bound and secretory protein. The enzymatic activity was shown by its digesting nucleic acid and NETs matrix, binding and internalizing macrophages and then causing cytotoxicity and apoptosis. Therefore, this is the first report to systematically characterize an *M. bovis* nuclease.

### 3.1. rMbovNase Is a Membrane Bound and Secretory Nuclease

The secreted proteins are usually associated with virulence factors such as antigenic or toxic proteins which can mislead the host immune response or damage the colonized tissue. A few mycoplasma proteins are recognized as secretory proteins, such as *M. hyopneumoniae* P102 [18], *M. fermentans* lipoprotein MALP-404 [19], and *M. hominis* P80 [20]. These proteins carry a type I signal sequence. Through the uncleaved signal peptide, they are surface-exposed and membrane anchored, while after signal peptidase I cleavage, they are released from the membrane into the supernatant as the secreted proteins. This phenomenon was summarized as the amphoteric model for mycoplasma secretory proteins which may be one of the solutions to a mycoplasma life with a reduced gene set. For *M. bovis*, to our knowledge, it is the first description of secreted protein with a type I signal sequence that is both embedded in the membrane and released from the membrane into the supernatant.

Its nuclease activity was solidly confirmed by its ability to degrade host DNA and RNA and plasmid DNA. Generally speaking, the degradation of host chromosomal DNA by microorganisms might be an approach to provide the precursors for its growth and survival [4,21]. Similar to *M. genitalium* MG_186 [9], rMbovNase showed a preference for RNA rather than DNA shown by the rapid and high degradation efficiency. It might be useful for mycoplasma to suppress host cell metabolism at RNA level. In addition, the temperature range of rMbovNase activity is wide in the presence of Ca^2+^. Since *M. bovis* has an extensive distribution *in vivo* from conjunctiva, ears, upper respiratory tract and lungs, joints, and other organs and tissues [22,23,24], this wide temperature adaptation would benefit mycoplasma survival, especially in thermoregulated tissues [9]. Because cellular mitochondrial uptake of Ca^2+^ during ATP synthesis contributes to generation of reactive oxygen species (ROS) [25], the Ca^2+^-dependent MbovNase nuclease activity might take Ca^2+^ competitively with host cells thereby inhibiting host oxidative defense [26]. Coincidently, *in silico* analysis has shown that the MBOV_RS02825 gene is located upstream of ABC transporters, similar to the endonuclease genes of *M. hyopneumoniae* mhp379 [5] and *M. agalatiae* MAG_5040 [27], the putative ABC transport system might facilitate import of degraded products of MbovNase, such as nucleotide precursors, into the mycoplasma cell [28].

In response to detrimental stimulation, such as bacterial infection, NETs structures may be generated. It has been shown that NETs could always entrap Gram-positive and -negative bacteria, as well as fungi, as part of host defense [29,30]. In this study, treatment of fresh isolated neutrophils with NETs stimulators (PMA and *M. haemolytica*) triggered NETs formation. However, we found that even at a high MOI *M. bovis* it could not stimulate NETs formation by bovine neutrophils (Figure 3). As reported, the prerequisite for ET information is the membrane rupture of host cells [31]. Whether the reason that *M. bovis* does not induce NETs is associated with the non-rupture of the cell membrane remains to be investigated in the future.

On the other hand, the microorganisms captured by NETs might fight against being killed by disruption of NETs by their DNase [32,33]. Some bacteria were reported to produce extracellular nucleases that can disrupt the DNA matrix of NETs, such as GBS0661 [34], SsnA [35], and EndAsuis [36]. We firstly confirmed that the purified rMbovNase could degrade bovine NETs and that this function might be determined by the TNASE_3 domain. *In vivo*, the co-infection of *M. bovis* with other bacteria is very common, like *M. haemolytica* [37]. This bacteria could induce NETs by leukotoxin in a CD18-dependent manner [38]. We speculate that MbovNase might help other bacteria such as *M. haemolytica* escape from NETs capturing and result in infection. Beside the form of membrane bound MbovNase, the secretory MbovNase might provide more convenient conditions to degrade NETs.

The purification of this 6×His tags protein with nickel affinity resin might generate some unfolded or misfolded target proteins due to high concentrations of denaturant, and the protein at high concentration (about 2 mg·mL^−1^) under frozen storage might form a soluble aggregate in solution. Both scenarios might affect its physiological solution behavior to a certain degree. Therefore, removal of any unfolded or misfolded protein could increase the specific activity of this purified protein in subsequent enzymatic assays.

### 3.2. Binding, Internalization, and Cytotoxicity of rMbovNase

The initial steps of interaction between *M. bovis* and host cells are adherence and subsequent internalization [39]. Many membrane proteins provide portals for this process [40]. Thereby the membrane-associated MbovNase is beneficial to binding and invasion. In the macrophage model, we determined that rMbovNase could specifically bind and internalize the cells and translocate into the nuclei. The nuclear localization could help the nucleases to target their substrates and cause DNA damage. A previous report had found nucleases can translocate from mitochondria to the nucleus and cleave chromatin DNA into fragments during apoptosis [41]. Moreover, *M. genitalium* similar to other bacteria like *Mycobacterium tuberculosis* and *Shigella flexneri*, could also deliver some enzymes like nuclease into the host cell nucleus and alter cellular function by modulating signal transduction pathways [42,43,44].

The ability of rMbovNase variant without TNASE_3 region exhibited deficiency in enzymatic activity, cellular binding, internalization and nucleus translocation. This is in agreement with the previous study [7]. The staphylococcal nuclease (SNc) domain homologous to TNASE_3 has been implicated in binding to RNA or single-stranded DNA and hydrolyzing DNA and RNA [45]. The mechanisms for protein binding, and thereby internalizing, vary greatly. Some reports demonstrated lysine-rich and other polar amino acid-rich regions in proteins are associated with binding and internalization [46,47,48]. In mycoplasma species, only *M. pneumoniae* nuclease Mpn133 contains this kind of EKS region with a lysine-rich domain which contributes to its cell binding and internalization properties [6]. Since we did not find any lysine-rich and other polar amino acid-rich regions in *M. bovis* MbovNase, it would be an alternative mechanism to mediate MbovNase binding and internalization.

A previous report has found that mycoplasma endonuclease isoforms could induce apoptosis [49]. In the current study, we demonstrated rMbovNase could induce apoptosis of bovine macrophage cell line BoMac cells. This phenomenon was supported by up-expression of apoptotic signal molecules such as phosphorylated NF-κB p65 and Bax and down-expression of apoptosis inhibiting signal molecules such as Bcl-2. Therefore, rMbovNase induced apoptosis might be cell dependent. Furthermore, this apoptosis is dependent on the TNASE_3 domain.

## 4. Materials and Methods

### 4.1. Ethics Statement

Animal experiments followed the Hubei Regulations for the Administration of Affairs Concerning Experimental Animals issued from 2005. They were approved by Hubei Province Science and Technology Department, which is responsible for experimental animal ethics (Permit Number: SYX-K (ER) 2010-0029 issued on 27 May 2010), and were supervised by the experimental animal ethics committee of Huazhong Agricultural University.

### 4.2. Bacterial Strains, Plasmids and DNA Manipulation

The *M. bovis* HB0801 strain used in this study is maintained at the China Center for Type Culture Collection (CCTCC no: M2010040), Wuhan. It was isolated from Hubei, China in 2008 at our laboratory. The strain was grown in pleuropneumonia-like organism medium (BD Company, Sparks, MD, USA), as previously described [50]. *E. coli* strains DH5α and BL21 (TransGen Biotech, Beijing, China) were grown in Luria–Bertani broth and used to clone the gene MBOV_RS02825 and expressed the recombinant *M. bovis* nuclease (rMbovNase). The pET30a His–tag, expression vector (Novagen, Darmstadt, Germany) was used for DNA manipulation. The *M. haemolytica* strain was isolated at our laboratory from a bovine lung lesion [51] and grown in tryptone soy broth or tryptone soy broth agar (BD company) at 37 °C.

### 4.3. Cell Culture Conditions

A BoMac cell line was kindly provided by Judith R. Stabel from the Johne’s Disease Research Project at the United States Department of Agriculture in Ames, Iowa, and grown as described previously [52]. Neutrophils were isolated from peripheral blood of healthy cattle by Ficoll–Hypaque gradient centrifugation (Hao Yang, Tianjin, China) according to the manufacturer’s instructions and had a purity of over 90%.

### 4.4. Computer-Assisted Sequence Analysis

The MBOV_RS02825 sequence (old_locus_tag = “Mbov_580”) of *M. bovis* HB0801genome (accession number: AFM51934.1) was retrieved from the NCBI database [53] and analyzed using on line PROSITE database [54]. The alignment of amino acid and nucleotide sequences between *S. aureus* SA_NUC (accession number: EFG57831) and MBOV_RS02825 was performed with online BLASTP [55]. The signal peptide cleavage sites, its type of signal peptidase and transmembrane helices in MbovNase were predicted by using online Signal IP, LipoP 1.0 and TMHMM 2.0 server [56].

### 4.5. Cloning and Expression of MBOV_RS02825 and Generation of Antiserum

Total mycoplasma DNA was extracted from TaKaRa MiniBEST Bacteria Genomic DNA Extraction Kit (TaKaRa, Dalian, China). After changing TGA to TGG at six sites within the MBOV_RS02825 gene using specific primer sets from 580 F1/R1 to F6/R6 to ensure tryptophan was encoded in *E. coli* (Table 1), the intact MBOV_RS02825 devoid of the lipoprotein signal sequence was amplified and subsequently cloned into pET30a to obtain pET30a–MBOV_RS02825 (363 aa) encoding His-tagged rMbovNase. Then, pET30a-MBOV_RS02825 was transformed into *E. coli* BL21 and rMbovNase was expressed by 0.8 mM IPTG induction at 37 °C for 2 h, purified by nickel affinity chromatography under native conditions, and eluted with lysis buffer containing 500 mM imidazole. Finally, buffer-exchanged to PBS in a 30 kDa Amicon Ultra-15 centrifugal filter unit (Millipore) and final preparation was stored in PBS (pH 7.4, 10 mM) at −80 °C until used.

To produce the variant MbovNase^Δ181–342^ without the TNASE_3 region at aa 181–342, a 544–1026 nt DNA fragment of MBOV_RS02825 was deleted by overlapped extension PCR with specific primers (Table 1). The mutated gene was cloned, expressed, and the rMbovNase^Δ181–342^ was purified as described above.

rMbovNase antiserum was produced by immunizing five female BALB/c mice at 4 weeks of age. Mice were primed with rMbovNase 100 μg in 250 μL and an equal volume of Freund’s complete adjuvant and boosted twice with the same doses of protein and Freund’s incomplete adjuvant at 2-week intervals. After the secondary immune response, serum samples were collected and used for immunological characterization.

### 4.6. Analysis of rMbovNase Nuclease Activity

Nuclease activity of rMbovNase was analyzed by agarose gel electrophoresis as described elsewhere [26]. Briefly, 2.5 μg of rMbovNase was incubated with 1 μg three nucleic acid substrates, respectively. BoMac cellular DNA was extracted by TissueGen DNA kit (Cwbiotech, Beijing, China) and RNA, and closed circular plasmid DNA at 37 °C for various times in 50 μL of nuclease reaction buffer (100 mM Tris–HCl, pH 8.5) containing 10 mM CaCl_2_. At predetermined times, 10 μL of reaction mixture was removed and 0.1 μL 1 M EDTA was added to terminate the reaction. The digested products were visualized by 1% agarose gel electrophoresis. The optimal temperature for nuclease activity within the range of 22–65 °C was determined using plasmid DNA as the substrate. rMbovNase (2.5 μg) was co-incubated with different concentrations of metal ions including CaCl_2_, MgCl_2_, ZnCl_2_, MnCl_2_, NaCl, and KCl, and the effect on nuclease activity was evaluated as described above. The nuclease activity of rMbovNase^Δ181–342^ was also assayed using plasmid DNA in parallel, and PBS was used as the negative control. The nucleic acid concentrations were read at 260 nm using a Nano-Drop2000 (Thermo Fisher, Rockford, IL, USA), and the proteins were quantitated by the bicinchoninic acid (BCA) method (Thermo Fisher).

### 4.7. Zymogram Analysis

Zymogram analysis involves inhibition of nuclease activity by sodium dodecylsulfate (SDS) and renaturation following SDS removal by diffusion [10]. Briefly, total cellular proteins of *M. bovis* HB0801, rMbovNase, or rMbovNase^Δ181–342^ protein were mixed with SDS loading buffer, heated to 100 °C for 10 min, and loaded onto 12% SDS-PAGE gels saturated in advance with herring sperm DNA (160 μg·mL^−1^; Sigma–Aldrich, St. Louis, MO, USA). After electrophoresis, the proteins on gels were allowed to recover their activities in renaturation buffer (40 mM Tris–HCl, 1% skimmed milk, 0.04% β-mercaptoethanol, 2 mM CaCl_2_, 2 mM MgCl_2_) at 37 °C for 8 h. DNA was visualized by ethidium bromide staining. Nuclease activity was identified by the presence of a specific, clear band at the site of rMbovNase resulting from DNA hydrolysis. Band size was estimated using prestained 10 to 180 kDa markers (Thermo Fisher, Rockford, IL, USA).

### 4.8. NETs Formation and Degradation Assay

To test whether *M. bovis* could induce NETs, neutrophils (2 × 10^5^ in 500 μL) were seeded on coverslips in a 24-well plate and stimulated with *M. bovis* at different MOI (10, 100, 1000) for different times (1, 2, 3, and 4 h), while *M. haemolytica* (2 × 10^7^ CFU in 100 μL) [38], or 200 nM PMA (Sigma) as the positive control to induce NET formation as previously described [57]. The plate was placed in a humidified incubator at 37 °C with 5% CO_2_ for 4 h and then the cells were fixed with 4% paraformaldehyde (PFA) for 10 min. NETs were stained with DAPI for 5 min and observed with a fluorescence microscope (Nikon Eclipse 80i, Tokyo, Japan). After NETs formation as described above, either rMbovNase or rMbovNase^Δ181–342^ were added to each well in a 24 well plate and incubated for an additional 90 min. DNase I at 40 units per well (TaKaRa) was used as a positive control, PBS was a negative control, and untreated cells were a blank control. The degradation of NETs was visualized as described above.

### 4.9. Localization of rMbovNase by Western Blot Analysis

Membrane and cytoplasmic protein fractions were extracted with a Proteo-Extract Trans-membrane Protein Extraction Kit (Novagen) and total cell protein was isolated by disrupting *M. bovis* with a hydraulic homogenizer (JNBIO, Guangzhou, China). The protein concentration was determined by BCA method, 50 ng of the proteins were separated on SDS-PAGE, and western blots were performed as described previously [58]. Briefly, mouse antiserum against rMbovNase (1:500) and HRP-conjugated goat anti-mouse IgG (1:5000; Southern Biotech, Birmingham, MI, USA) were sequentially overlaid, and visualized by using an ECL substrate kit (Thermo Fisher). Purified rMbovNase and its variant (2 ng in 10 μL) were used as positive controls.

In order to determine whether MbovNase was a secretion protein, we detected the extracellular fraction from *M. bovis* growth medium. Briefly, 10 mL log-phase culture *M. bovis* in PPLO broth medium was centrifuged at 10,000× *g* for 30 min to obtain the supernatant, then the supernatant was concentrated to 500 μL by ultrafiltration. The blank medium was taken as a negative control. 10 μg of proteins were loaded and subjected to SDS-PAGE and analyzed with western blot as described above.

### 4.10. rMbovNase Binding and Invasion Assay

BoMac cells were inoculated onto glass coverslips at a density of 1 × 10^5^ cells and propagated for 24 h. Then 10 μg·mL^−1^ of rMbovNase or rMbovNase^Δ181–342^ was added to the cells for 30 min at 4 °C in the binding assay, or for 24 h at 37 °C in the invasion assay. The cells were washed with PBS and fixed with 4% PFA. In the invasion assay, BoMac cells in PBS were permeabilized by 0.1% Triton X-100 for 10 min. Then, in both the binding and invasion assays, cells were immunolabeled with mouse anti-rMbovNase antibody and goat anti-mouse lgG(H+L)-FITC (Southern Biotech, Birmingham, AL, USA). The nuclei were counterstained with DAPI (Beyotime, Shanghai, China); cytoplasmic actin filaments were counterstained with rhodamine phalloidin (Cytoskeleton, Denver, CO, USA). The slides were coverslipped and observed by confocal laser fluorescent microscopy (Olympus FV1000 and IX81, Tokyo, Japan). In order to determine quantitative differences in binding and internalization by the wild-type and its variant, we used flow cytometry detection and IPP6.0 software analysis, respectively. Flow cytometry assay was used to determine rMbovNase binding ratios. Briefly, BoMac cells (1 × 10^6^) were incubated with 10 μg·mL^−1^ rMbovNase or rMbovNase^Δ181–342^ at 4 °C for 30 min in 10% goat serum in PBS, followed by incubation with mouse antiserum to rMbovNase (1:500), and goat anti-mouse lgG(H+L)-FITC (1:1000, Southern Biotech). The cells were analyzed for binding efficiency after washing and resuspending in 300 μL PBS, using a fluorescence-activated cell sorter (Becton Dickinson, San Jose, CA, USA). For the invasion assay, the cells were treated as described above and observed under a confocal microscope. A total of about 100 cells from 10 fields in each sample were analyzed. The positive cells were defined by five or more puncta overlaid with the nucleus in the cells. Using IPP6.0 software analysis, the percentage of positive cells protein nuclear localization was determined by counting the number of BoMac cells with protein-related immunofluorescent punctuate inside individual nuclei and dividing by the total number of cells. The location of rMbovNase or rMbovNase^Δ181–342^ protein within the individual nuclei was also observed by evaluating Z-series data stacks using the multiplane form function of Fluoview software (Olympus FV1000 and IX81, Tokyo, Japan). Z-series datasets were generated at 1 μm interval between the basal to apical side of the cell monolayer using samples prepared at 24 h post-stimulation.

### 4.11. MTT Assay of Cell Viability after rMbovNase Treatment

BoMac cells were seeded in a 96-well plate at 1 × 10^4^ cells per well, incubated overnight and treated with rMbovNases, rMbovNase^Δ181–342^ or PBS for 24 h at 37 °C. Wells without cells were used as blank controls. MTT solution (Vazyme, Nanjing, China) was added to the wells for additional 4 h at 37 °C, then the supernatants were discarded, and DMSO 100 µL was added into each well to dissolve the intracellular formazan precipitate resulting from MTT reduction. The OD_570nm_ of each well was measured, and the viability (%) was calculated as the ratio of (OD_sample_ − OD_blank_)/(OD_PBS_ − OD_blank_).

### 4.12. Detection of Cell Apoptosis Induced by rMbovNase

BoMac cells were cultured in six-well plates (Corning, New York, NY, USA) and exposed to either rMbovNase or rMbovNase^Δ181–342^ at concentrations of 0.25, 0.5, or 1.0 µM for 24 h. Apoptotic cells were labelled with an annexin V-FITC/PI apoptosis detection kit (KeyGEN BioTech, Nanjing, China), and assayed with flow cytometry. Apoptosis stimulated with the inducer in Apoptosis Inducers Kit (Beyotime, Beijing, China) was used as the positive control.

### 4.13. Western Blot Assay of Molecular Expression Related to Apoptosis and the NF-κB Signal Pathway

Western blot analysis was carried out to confirm that rMbovNase induced phosphorylated NF-κB signal transduction and apoptosis-related molecules. BoMac cells (1 × 10^6^ per well) were incubated with either 1 µM rMbovNase or rMbovNase^Δ181–342^ at 37 °C for 24 h, washed, and suspended in 200 μL RIPA cell lysis buffer (Beyotime) for 1 h at 4 °C. Lysates were centrifuged at 10,000× *g* for 10 min at 4 °C and the supernatants were collected. Total protein concentration was determined with a BCA protein assay kit (Thermo Fisher). Aliquots of 50 ng in SDS loading buffer were separated on a 12% SDS-PAGE gel and transferred to a polyvinylidene fluoride membrane (Millipore, Billerica, MA, USA). After blocking with 10% goat serum in TBST buffer, the membranes were incubated at 4 °C overnight with primary antibodies including rabbit polyclonal antibodies to Bax, Bcl-2, NF-κB p65, NF-κB phospho-p65, Iκβα, phospho-Iκβα, ERK1/2, phospho-ERK1/2 and β-actin (Cell Signaling Technology, Danvers, MN, USA) each diluted to 1:2000 with blocking buffer. After washing with TBST buffer, the membranes were incubated with goat anti-rabbit IgG-HRP (1:3000) for 1 h at room temperature. The labeled proteins were detected using enhanced chemiluminescence reagents (Thermo Fisher). β-Actin was used as the internal reference.

### 4.14. Statistical Analysis

Each treatment was carried out in triplicate and all experiments were performed independently at least three times. Data were expressed as means ± SD, and the statistical analyses were conducted with GraphPad Prism version 5 (La Jolla, CA, USA). Differences were considered as statistically significant when *p* < 0.05 (*) and very significant when *p* < 0.01 (**) or *p* < 0.001 (***).

## 5. Conclusions

This study confirmed that rMbovNase is a membrane-bound and secretory nuclease. The nuclease activity was shown by degrading cellular DNA, and RNA and plasmid DNA and NETs in an RNA preference. In addition, it can bind, internalize the cells and localize in both cytoplasma and nuclei, induce apoptosis, and reduce cellular viability of bovine macrophages. These multiple functions dependent on TNASE_3 domain provide a potential mechanism of *M. bovis* cytotoxicity.

## Figures and Tables

**Table 1:** Oligonucleotide primers for PCR used in this study.

Names	Primer Sequences (5′-3′)	Positions
MBOV_RS02825		
580F1	CGGGGTACCGAAAA**TGG**CACAATTAAG	79→97
580R1	AGTTTTTTCTCCCAG**CCA**TCTACTAACTC	316←345
580F2	GTTAGTAGATGGC**TGG**GAGAAAAAACTTAG	317→347
580R2	AATATTTAGCCTTCAATTTGTC**CCA**ATCTATG	437←469
580F3	CTAAATATTTTGATGCAGAAATAGTTAAA**TGG**AGCG	460→496
580R3	GGCATAATGCTC**CCA**GTAAACTGGATTTTT	855←885
580F4	AATCCAGTTTAC**TGG**GAGCATTATGCC	855→885
580R4	TAAATGTTAGATTGAAT**CCA**GTATGGC	956←983
580F5	GCCATAC**TGG**ATTCAATCTAACATTTA	956→983
580R5	AGTGTTTATCTAGCAATGT**CCA**TTTTC	1000←1027
580F6	TGGAAAA**TGG**ACATTGCTAGATAAACA	998→1025
580R6	CGCGGATCCTTATTTATTTTTGTATGAATC	1071←1092
MBOV_RS02825^ΔTNASE_3^		
580F1	CGGGGTACCGAAAA**TGG**CACAATTAAG	79→97
580UR	TGGCAATGCAAATTTAGCCTTCAATTT	530←543, 1027←1040
580DF	AAATTGAAGGCTAAATTTGCATTGCCA	530→543, 1027→1040
580R6	CGCGGATCCTTATTTATTTTTGTATGAATC	1071←1092

The underlined sequences were sites for restrictive digestion. The bold indicates TGA to TGG change to permit tryptophan expression in *E. coli*. The arrows showed nucleotide position within MBOV_RS02825 coding open reading frame (ORF).

## Supplementary Materials

Supplementary materials can be found at http://www.mdpi.com/1422-0067/17/5/628/s1.
