# Supplementary material for: Mycoplasma bovis MBOV_RS02825 Encodes a Secretory Nuclease Associated with Cytotoxicity
Source: Int J Mol Sci. 2016 Apr 29;17(5):628. doi: 10.3390/ijms17050628 (PMC4881454; doi:10.3390/ijms17050628)
Supplement: Supplementary file 1 [file ijms-17-00628-s001.pdf]

Int. J. Mol. Sci. **2016**, *17*, 628; doi:10.3390/ijms17050628

Supplementary Materials: *Mycoplasma bovis* MBOV_RS02825 Encodes a Secretory Nuclease Associated with Cytotoxicity

Hui Zhang, Gang Zhao, Yusi Guo, Harish Menghwar, Yingyu Chen, Huanchun Chen and Aizhen Guo

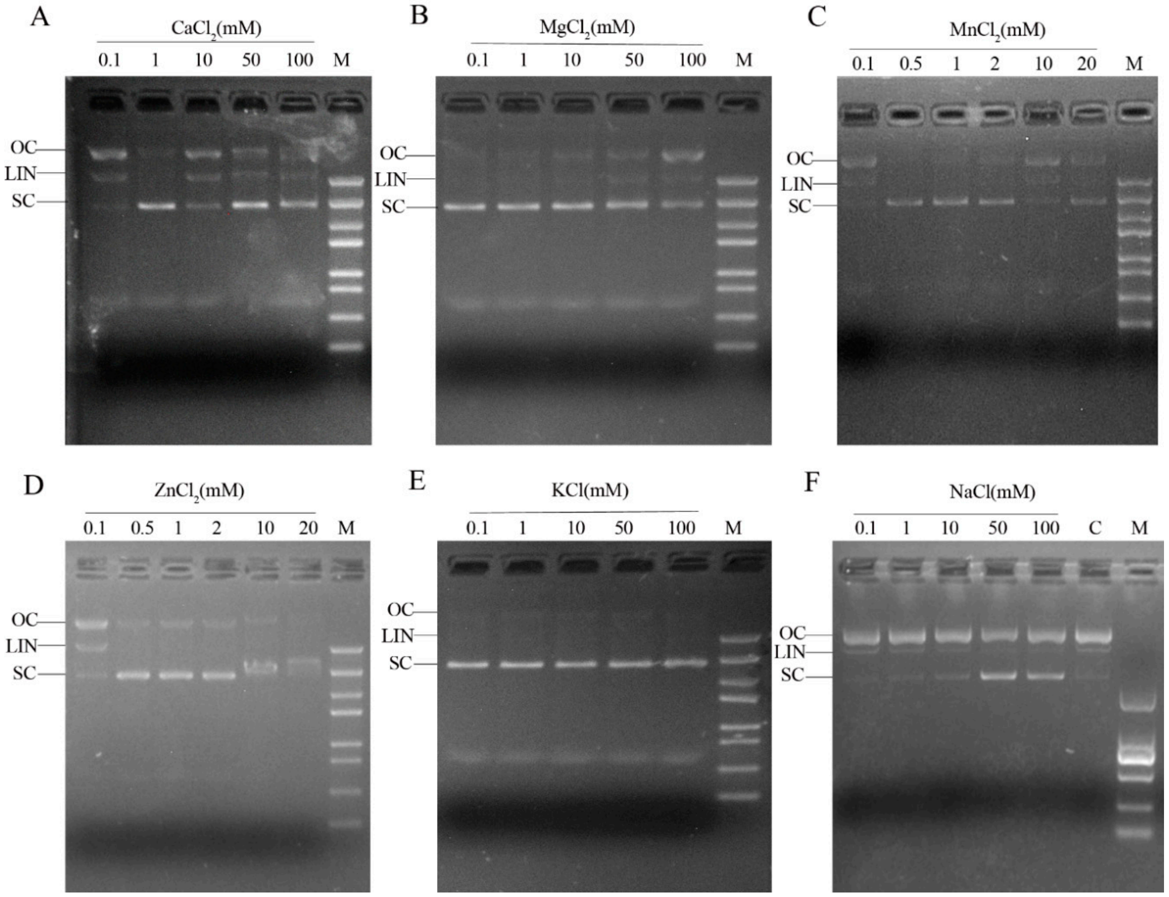

Figure S1. Effect of metal ions on rMbovNase activity. (**A**–**F**) represent effect of $CaCl_2$, $MgCl_2$, $MnCl_2$, $ZnCl_2$, KCl, and NaCl presence on rMbovNase activity respectively. For all the groups, plasmid DNA (1 μg) was incubated with rMbovNase in the presence of $CaCl_2$, $MgCl_2$, $MnCl_2$, $ZnCl_2$, KCl, and NaCl at different concentrations from 0.1 to 20 mM indicated above each lane and the digestion effects were compared. Reactions were stopped by adding EDTA solution. Test samples were resolved on 1% agarose gel. Open-circle (OC), linear (LIN) and supercoiled (SC) forms of plasmid DNA are indicated on the left of each picture. The untreated with any metal ions was negative control (Lane C).
